# Occupational Ocular Injuries and Utilization of Eye Protective Devices among Sawmill Workers in the Ojo Local Government Area of Lagos State, Nigeria

**DOI:** 10.3390/vision5040060

**Published:** 2021-12-09

**Authors:** Ngozika E. Ezinne, Kingsley K. Ekemiri, Maryann A. Nwanali Daniel

**Affiliations:** 1 Department of Clinical Surgical Sciences, Saint Augustine Campus, University of the West Indies, St. Augustine 999183, Trinidad and Tobago; Kingsley.ekemiri@sta.uwi.edu; 2 Department of Optometry, Madonna University Elele, Elele 5001, Rivers State, Nigeria; maryamarachi46@yahoo.com

**Keywords:** occupational ocular injuries, utilization of eye protective devices, sawmill workers, Nigeria

## Abstract

In this work, we carried out a cross-sectional study to assess occupational ocular injuries and utilization of eye protective devices among sawmill workers in the Ojo local government area of Lagos State, Nigeria A structured questionnaire was used to conduct face-to-face interviews among the sawmill workers. Pearson’s chi-squared test and *t*-test were used to test associations between variables. A *p*-value of less than 0.05 was considered statistically significant. A total of 215 sawmill workers with a mean age of 37.08 ± 12.07 years participated in the study. A majority (55.8%) of the participants were male (93.7%), and a majority were 21–40 years old (55.8%). Of the participants, 78.6% were aware of occupational ocular injuries and 17.7% used ocular safety devices. The major barrier to the use of eye protective devices was unavailability (43%). Workers who were ≥20 years old (*p* < 0.001), who received a monthly salary of less than USD 100 (*p* < 0.043), who had work experience of ≥10 years (*p* < 0.04), who were aware of ocular hazards (*p* < 0.03), and who did not use protective eye devices (*p* < 0.02) were significantly associated with occupational ocular injuries compared to others. The prevalence of occupational ocular injuries and the utilization of eye safety devices among the sawmill workers in the current study were comparable to findings from other studies. Based on the results of our study, we advise the provision of ocular protective devices for sawmill workers and policies to enforce regular utilization.

## 1. Introduction

Occupational injuries represent 8% of all unintentional injuries and result in more than 3.9 million deaths worldwide; they are particularly prevalent in low- and middle-income countries [1]. Approximately 10–20% of the gross national product is lost each year as a result of 270 million work-related injuries and 160 million occupation-related diseases [2]. Moreover, work accidents are a frequent cause of eye injuries and are responsible for 30–70% of admissions to ophthalmological emergency departments [3].

Globally, about 2.5 million people suffer eye injuries annually and more than 500,000 injuries resulting in blindness take place each year [4]. Work-related eye injuries constitute a public health problem, being responsible for many significant cases of morbidity and making up a substantial proportion of all work-related injuries [5]. Moreover, they are considered to be largely preventable, especially if adequate eye protection is used regularly [4].

A sawmill is a facility or factory where raw timber or logs of timber are processed and sawn into planks or boards by a machine [6]. They generate a lot of environmental pollutants, including sawdust, falling debris, flying objects (both wood and metals), chemicals used for wood processing and preservation, electric shocks, and various types of fuel and gaseous emissions that are harmful to health and the visual system [6,7,8]. Over the years, sawmill operations have changed significantly in developed countries to reduce waste, increase energy efficiency, and improve operator safety [9]. The once-ubiquitous rusty, steel conical sawdust burners have, for the most part, vanished, and sawdust and other mill waste are now processed into particleboard and wood chips used for paper mills and strand board paneling for building construction. Such changes have also led to increases in profits [9]. However, in Nigeria, due to a lack of funds, the old sawmill systems are still used and workers still operate in conditions detrimental to their health due to exposure to sawdust and chemicals used in wood treatment [6,10].

Safety precautions, including utilization of eye safety devices among workers, especially in small-scale industries in Nigeria, are not routinely enforced nor practiced. Occupational injuries are not reported; hence, data on occupational injuries are not available [10]. Occupational ocular injuries among sawmill workers have been reported in studies of other places; there are no recent studies for Lagos State, Nigeria. There is a great need to study the conditions of sawmill workers to determine the prevalence and severity of occupational injuries, as well as rates of ocular safety device use. The data generated from this study will provide useful information that could be used to develop policies and strategies for promoting eye health and preventing sawmill-related ocular injuries. The study would also provide practical insights into planning strategies, such as education and safety training for sawmill workers, in order to reduce ocular injuries associated with sawmill work. Therefore, this study aimed to determine the prevalence of occupational ocular injuries and utilization of eye safety devices among sawmill workers in the Ojo local government area of Lagos State.

## 2. Materials and Methods

### 2.1. Study Setting

This study was carried out in the Ojo local government area of Lagos State, Nigeria. Ojo is one of the 20 local government areas in Lagos State, with a population of 507,693 [11]. The LGA has a total land mass of 180 sq. km, about 30% of which constitutes the riverine area [11]. Like other parts of the Lagos metropolis, it is made up of a complex mix of people from various parts of Nigeria. The majority of the residents are civil servants or artisans, including sawmillers, craftsmen in woodwork, and welders. The Ojo local government area (LGA) was selected for the study due to the heterogeneity of its residents and its geographical location.

### 2.2. Study Design

This was a cross-sectional descriptive study of all sawmill workers in Ojo to assess the prevalence of occupational ocular injuries and utilization of ocular protective devices.

### 2.3. Sampling Technique

There are no data on the total number of sawmills in Ojo. Therefore, five identified sawmills with a total of 230 workers were chosen for the study. Those who had worked for less than 6 months in the identified sawmills and those who failed to give their consent to participate in the study were excluded.

### 2.4. Ethical Consideration

Ethical clearance was obtained from the Ethics and Research Committee of the Madonna University, Elele campus, Rivers State. The research adhered to the Declaration of Helsinki on research regarding human subjects. Permission to conduct the study in the identified sawmill was obtained from the relevant authorities. Verbal consent to participate in the study was obtained from all eligible sawmill workers.

### 2.5. Data Collection Procedure

The study was conducted over a 6-month period, from July to December 2019. Sawmills in Ojo were identified and visited. We met with the managers of the sawmills and explained the purpose of the study to them. Appointments were scheduled to meet with the workers to invite them to participate in the study. An ocular health talk was organized for the workers prior to data collection. Moreover, information about the study was provided and the procedures and importance of participating in the study were explained to the workers. Those who gave their consent to participate in the study were included in the study. The data were collected using a pretested structured questionnaire that was administered to all workers who gave their consent to participate. The questionnaire had items on socio-demographic characteristics, workplace characteristics, ocular injuries, ocular complaints, use of protective eye devices, and barriers to use of such devices. A pre-test, or pilot, was conducted among 10 (5% of 215) sawmill workers in Lagos Island, outside the study area. After the pre-test, the necessary modifications were made to the questionnaire and the data were collected through face-to-face interview by four optometrists.

All ocular examinations were carried out during the day at the sawmills. Assessment of visual acuity (VA) for each eye was performed using the Snellen chart (Illiterate E-chart), which was placed outdoors at a 6 m distance from the participant. Each eye was tested separately, unaided, and further testing with a pinhole was performed in cases where visual acuity was less than 6/6. Visual acuity in the better eye of 6/6–6/12 was considered to be normal, 6/18–6/60 was classified as visual impairment, <6/60–3/60 was classified as severe visual impairment, and visual acuity less than 3/60 was classified as blindness.

The visual field was assessed in each respondent using the confrontational method compared with examiners. The test was conducted by the examiner and the patient sitting directly facing each other at eye level and closing the eyes that were directly facing each other. The patient was asked to fixate on the open eye of the examiner, and a 5 mm red-headed pin was moved inward from beyond the boundary of each quadrant along a line bisecting the horizontal and vertical meridians. The patient was asked to report when the pin was first perceived to be red. The same procedure was repeated for the other eye. The examiner performed an automated perimetry to confirm a normal central visual field prior to the data collection. Any deviation from the examiner’s field was recorded as abnormal. Broad H tests were conducted to check extra-ocular muscle activity, tested in all directions of gaze, seeking for any paresis or paralysis of the extra-ocular muscles. Diplopia was sought in all directions of gaze.

External eye examinations and anterior segment examinations were performed using a pen torch. The presence of tarsal papillae, pterygium, pinguecula, and conjunctival congestion was noted. The presence of corneal lesions and opacities was noted. Corneal lesions were stained with fluorescein and examined with the pen torch (only the superficial layer stained was noted because of the use of the pen torch). Crystalline lens and the fundus were examined using a direct ophthalmoscope (Keeler ophthalmoscope).

### 2.6. Operational Definition

#### 2.6.1. Occupational Ocular Injury

Here, we define occupational ocular injury as any eye injury that occurred to the worker while working in a sawmill industry within the last 12 months.

#### 2.6.2. Awareness of Eye Safety Devices

Awareness of ocular safety devices includes knowledge of ocular safety devices and their usefulness in protecting workers against sawmill-associated ocular hazards.

#### 2.6.3. Awareness of Occupational Ocular Hazards

Awareness of occupational ocular hazards implies knowledge of potential hazards to the eye associated with sawmill work and sawdust exposure.

#### 2.6.4. Ocular Safety Devices

Here, we consider ocular safety devices as equipment such as goggles, face shields, and helmets designed to protect the eyes from hazards faced by sawmill workers.

#### 2.6.5. Utilization of Ocular Safety Devices

Utilization (regular use) of ocular safety device is defined as wearing ocular safety devices during work or sawdust exposure.

#### 2.6.6. Formal Education

Formal education includes 6 years each of primary and secondary education and 3 to 6 years of tertiary education, depending on the cadre of training institutions and course of study.

### 2.7. Data Analysis

Data were entered into Microsoft Excel and exported to Statistical Package for Social Sciences (SPSS) version 21 for analysis. Descriptive data were analyzed using frequency and percentage frequency. Pearson’s chi-squared test and *t*-test were used to test associations between variables (demography, awareness of ocular hazards, use of ocular protective devices, and type of ocular protective device used). A *p*-value of less than 0.05 was considered statistically significant.

## 3. Results

### 3.1. Demographic Characteristics of the Study Participants

Demographic data of the study participants are presented in Table 1. Out of 230 sawmill workers, 215, with a mean age of 37.08 ± 12.07 years, gave their consent to participate in the study, giving a response rate of 93.5%. The majority (55.8%) of the participants were males (93.7%), and the majority were 21–40 years of age (55.8%). It is worth noting that 43 (20%) of the study participants had no formal education and less than 10% had tertiary education. The majority (62.3%) of them earned less than USD 100 per month.

### 3.2. Workplace Characteristics of the Study Participants

More than 20% (21.9%) of the study participants were machine operators and the majority (127, 59.1%) of them had less than 10 years of working experience. Most (55.3%) of the participants worked more than 7 h per day and were aware of the occupational ocular hazards (78.6%) at the sawmill but did not use eye protective devices (82.3%). The major barrier to the use of eye protective devices was unavailability (43%), and 190 (88.4%) did not have training on the use of eye protective devices at work (Table 2).

### 3.3. Ocular Characteristics of the Participants

The majority (77.7%) of the respondents had visual acuity of 6/6 to 6/12 and 45 (20.9%) had no ocular complaints. Pinguecula (34.4%) and pterygium (19.5%) were the major ocular conditions found (Table 3).

### 3.4. Ocular Injuries among the Participants

A total of 170 participants (79.1%) had ocular injury during the study period, and most of the ocular injuries were corneal abrasions (68.8%). The major sources of ocular injury were sand and sawdust particles (47.1%). Ocular injuries were mostly found among machine operators (25.9%). Of the participants who reported eye injuries, 61 (35.9%) went to an eye clinic/hospital and 58 (34.1%) went to a pharmacy for treatment (Table 4).

### 3.5. Factors Associated with Occupational Ocular Injuries

Workers who were ≥20 years old (*p* < 0.001), earned less than USD 100 per month (*p* < 0.043), had work experience of ≥10 years (*p* < 0.04), were aware of ocular hazards (*p* < 0.03), or did not use protective eye devices (*p* < 0.02) were significantly associated with having occupational ocular injuries compared to others (Table 5).

## 4. Discussion

### 4.1. Demographic Characteristics

In the present study, we assessed occupational ocular injuries and use of ocular safety devices among sawmill workers in Ojo, Lagos State, Nigeria, and found a large male majority, similar to findings of other studies [7,12,13,14,15] among sawmill workers and other artisans. Crafts such as sawmill work are naturally physically demanding jobs, and this could be a reason for the male dominance of the field.

The mean age of our study participants was 37.08 years, and the largest age group was 21 to 40 years. This indicates that most of our study participants were of a relatively young age. Similar findings were reported in other studies, in Benin City, Nigeria [12], and Kumasi, Ghana [13]. However, Bert et al.’s (2016) [16] study of cocoa farmers in Ghana had more participants who were 60 years and older. Sawmill work involves more physical strength than cocoa farming, which could explain the reason for the variations in the findings. Moreover, the study setting could influence the recorded age group; for example, our study was conducted in a commercial city, whereas the study in Ghana [16] was carried out in a rural area.

A sizeable proportion of participants in studies of sawmill workers in Nigeria [10,15] and cocoa farmers in Ghana [17] had no formal education, findings similar to our study. Professions such as sawmill and cocoa farming in Africa usually train through an apprenticeship; thus, formal education is not entirely necessary. Additionally, technical and vocational education programs in Nigeria are expensive and not adequately funded nor equipped with the basic facilities, which may discourage potential trainees from enrolling [15].

### 4.2. Ocular Characteristics of the Participants

Tearing was recorded as the major ocular complaint in our study, contrary to blurring of near/far vision reported in other studies in Lagos State, Nigeria [15], and Ghana [17]. The age group included and the nature of work could affect the types of ocular complaints, which explains the reason for the variations in the findings between our study and others.

Onyekwelu et al. [15] recorded refractive error, cataracts, and glaucoma as the most prevalent ocular conditions among carpenters in their study in Mushin, Lagos State, Nigeria, contrary to pinguecula, pterygium, and cataracts recorded in the present study. The older age group included in the Mushin study when compared with ours could be the reason for such variations. Moreover, even though cataracts are predominantly an age-related condition, prolonged exposure of sawmill workers to ultraviolet rays due to their outdoor work puts them at a high risk of developing cataracts [6,7,8].

Prolonged exposure to ultraviolet radiations, dust, wind, and chemicals associated with sawmill work was reported to be a risk factor for pinguecula and pterygium. This was also found in the current study and other studies in Nigeria [7,18].

Similar to our findings was the prevalence of uncorrected refractive error recorded as a result of presbyopia among welders in Ile-Ife, Nigeria [18]. Presbyopia is an age-dependent condition that can easily be corrected with glasses, and no study has linked early presbyopia to sawmill work.

### 4.3. Occupational Ocular Injuries

The prevalence of occupational ocular injuries recorded in the current study is similar to the 75% recorded in India [19] but lower than the 85% reported in a rural community in Nigeria [20]. However, it is higher than the 47.9% and the 31.4% recorded in Ghana [17] and Ethiopia [21], respectively, among welders. It is considerably higher than the 10.7% and the 20.7%, reported in Benin City, Nigeria [12], and New Zealand [22], respectively. Unavailability of eye safety devices, lack of health safety training, and differences in socio-economic status have been implicated as causes of occupational ocular injuries [7,20,23]. Poor knowledge on the adverse effects of sawmill activities could also cause occupational injuries. The low utilization of eye safety devices recorded in our study could be the reason for the high prevalence of occupational ocular injuries. A study in Ethiopia of small-scale workers showed that workers who do not use eye safety devices are more than 7 times more likely to face occupational ocular injuries than those who wear eye safety devices. Regular use of eye safety devices among sawmill workers should be enforced. The major sources of ocular injuries recorded in the current study were similar to findings recorded in Ghana [13].

The use of traditional medicine or herbal treatments for various ocular ailments is still popular in most African countries [17], and this was confirmed by the high number of people who resorted to the use of traditional medicine after sustaining an injury recorded in the current study and studies in Ghana [17,24]. Herbal treatment should be discouraged, as it could lead to visual impairment and sometimes permanent loss of vision. Ocular education of sawmill workers on the need to seek proper and appropriate eye care services can never be over-emphasized.

The awareness of occupational hazards and utilization of ocular safety devices in this study was lower than 90.6% [7,23] and 99.4% [14], respectively, recorded among welders. However, it was higher than the 67.5% recorded among carpenters in Mushin, Lagos State [15]. The high level of awareness of sawmill-related ocular hazards suggests that occupational eye health safety interventions to reduce sawmill-related ocular hazards should focus more on overcoming the identified barriers to the utilization of eye safety devices rather than on hazard awareness. The most common eye safety device used in our study was goggles, consistent with findings from other studies [14,15,25].

The utilization of eye safety devices recorded in the current study was lower than the 26.3% recorded among carpenters in Mushin, Lagos State [15]. It was also lower than the 27–3% and the 47.7% recorded among welders in Ghana [17,25] and Nepal [26], respectively. Higher radiation exposure and the projectile nature of the offending foreign body associated with welding could prompt welders to use eye safety devices more than other artisans, including sawmill workers. Eye injuries are largely preventable, especially if adequate eye protections are used regularly.

The unavailability, cost, and inconvenience of eye protective devices, as well as workers’ lack of knowledge on how to use them, an unfelt need for them, forgetfulness, anticipation of reduction in productivity, type of task, short duration of task, anticipated low risk of task, previous ocular injury, and time factors are some of the reasons reported in various studies for low utilization of eye protective device among artisans [15,17,27]. Similar findings were reported in our study, with unavailability of eye protective devices being the major barrier to their utilization among the surveyed workers. There is a great need to enforce and make protective devices available for workers.

Lack of proper training on the use of ocular safety devices was reported to be significantly associated with occupational ocular injuries in various studies in Ethiopia [21,28], India [29], and China [30]. A similar finding was recorded in our study.

Our study recorded goggles as the most-used ocular safety device, findings similar to other works [14,15,23,25]. The use of goggles could be popular because they are more affordable and readily available than other options.

### 4.4. Factors Associated with Occupational Ocular Hazards

The current study recorded a significant association between non-use of protective ocular devices and having occupation ocular injuries. Similar findings were recorded in studies in Nigeria [4], Ghana [17], and Nepal [31]. This means that sawmill workers who do not use any protective eye device are exposing themselves to ocular hazards.

Ocular injuries were found to be significantly high among machine operators in the current study, and this was similarly reported in various other studies [7,12,17]. In addition, a study in Ghana [17] found electric/arc welders to be at a higher risk of ocular injuries than gas welders because of the high temperatures and voltages generated during electric welding.

High income was reported to be significantly associated with having ocular injuries in studies in Ghana [17] and Kenya [32]. However, in the current study, reduced income was found to be associated with ocular injuries. Lack of knowledge on the importance of using ocular safety devices could be the reason for such variations. Policies should be developed for sawmill workers to ensure their regular utilization of eye safety devices to reduce occupational ocular injuries.

### 4.5. Limitations

The small sample size and the fact that the data were collected from one local government area in Lagos State limits the generalization of the findings to the entire population. Additionally, participants’ recall bias inherent in self-report surveys along with the reliability of their self-reporting and knowledge are additional issues presenting further limitations to data extrapolation. Moreover, limitations could have arisen from respondents’ possible misunderstanding of the questions despite our explanations. The fact that only descriptive analysis, with no investigations of the relations between parameters and low consideration of possible confounders, was used further limits the study. Despite the limitations of this study, the sample size used was enough to represent the sawmill worker population in the study area.

## 5. Conclusions

The findings of this study suggest that there is a high prevalence of occupational ocular injuries among sawmill workers in the Ojo local government area of Lagos State, Nigeria, due to the risky nature of the work. Therefore, we strongly encourage the provision of ocular protective devices for sawmill workers and the development of policies to enforce their regular utilization.

## Figures and Tables

**Table 1:** Demographic characteristics of the participants.

Variable	Frequency (*n*)	Percentage (%)
**Age**		
<20	12	5.6
21–40	120	55.8
41–60	72	33.5
61–80	11	5.1
**Gender**		
Male	200	93.7
Female	15	7
**Educational Background**		
Nil	43	20
Primary school	35	16.3
Junior secondary school	40	18.6
Senior secondary school	79	36.7
Tertiary	18	8.4
**Marital Status**		
Single	77	35.8
Married	133	61.9
Widow	1	0.5
Widower	1	0.5
Divorced	3	1.4
**Monthly income in Naira**		
30,000–50,000 (USD ≤ 100)	134	62.3
60,000–100,000 (USD > 100 to 199)	58	27
>100,000 (USD > 200)	23	10.7

**Table 2:** Workplace characteristics of the participants.

Variables	Frequency (*n*)	Percentage (%)
**Nature of work**		
Convertor	27	12.6
Wood loader	27	12.6
Sawmill supervisor	7	3.3
Administrator	19	9.8
Jackman	30	14
Machine operator	47	21.9
Saw technician	21	9.8
Sawyer	16	7.4
Sawdust packer	21	9.8
**Work experience in years**		
1−10	127	59.1
11−20	68	31.5
21−30	20	9.3
**Number of work hours per day**		
≤6	96	44.7
≥7	119	55.3
**Awareness of use of ocular safety** **devices and ocular hazards**		
Yes	169	78.6
No	46	21.4
**Use of ocular safety devices**		
Yes	38	17.7
No	177	82.3
Goggle	20	52.6
**Reason for not using ocular safety** **devices**		
Do not like to use	58	32.8
Unavailability	76	43
Slows down work	1	0.6
Unfelt need	45	25.4
Causes discomfort	1	0.6
**Training on the use of ocular safety devices**		
Yes	25	11.6
No	190	88.4
**Frequency of safety training at work**		
Never	171	79.5
Sometimes	26	12.1
Often	18	8.4

**Table 3:** Ocular characteristics of the participants.

Variables	Frequency (*n*)	Percentage (%)
**Visual acuity**		
6/6 to 6/12 (normal VA)	167	77.7
6/18 to 6/60	45	20.9
Less than 6/60	3	1.4
**Ocular complaints**		
Tearing	32	14.9
Itching	21	9.8
Discharge	12	5.6
Foreign body sensation	20	9.3
Photophobia	6	2.8
Blurring of vision (far or near)	30	14
Redness	19	8.8
Pain	30	14
No symptom	45	20.9
Ocular problems		
Pinguecula	74	34.4
Pterygium	42	19.5
Cataract	40	18.6
Cornea ulcer	4	1.9
Allergic conjunctivitis	2	0.9
Chalazion	1	0.5
Refractive error	40	18.6
Glaucoma	7	3.3
Bacterial conjunctivitis	3	1.4
Anterior uveitis	1	0.5
Internal hordeolum	1	0.5

**Table 4:** Ocular injuries among the participants.

Variables	Frequency (*n*)	Percentage (%)
**Have had ocular injuries**		
Yes	170	79.1
No	45	20.9
**Type of ocular injuries**		
Superficial conjunctiva or corneal foreign body	117	68.8
Blunt trauma	47	27.6
Lid injury	3	1.8
Laceration	3	1.8
**Source/cause of the injury**		
Machine	13	7.6
Sand and sawdust particles	80	47.1
Spray	10	59
Wood	14	8.2
Work tools	53	31.2
**Ocular injuries according to nature of work**		
Convertor	9	5.3
Wood loader	27	15.9
Sawmill supervisor	2	1.2
Administrator	8	4.7
Jackman	26	15.3
Machine operator	44	25.9
Saw technician	13	7.6
Sawyer	20	11.8
Sawdust packer	21	12.3
**Eye health seeking behavior after the ocular injury**		
Eye clinic/hospital	61	35.9
Pharmacy/drug store	58	34.1
Herbal medicine	21	12.4
Self-treatment	13	7.6
Did nothing	17	10

**Table 5:** Factors associated with occupational ocular injuries.

Variables	Ocular Injuries		*p*-Value
	Yes	No	
**Gender**			
Males	160	40	0.22
Females	10	5	
**Age**			
18–20	7	5	0.001
21–40	83	37	
41–60	69	3	
61–80	11	0	
**Number of hours worked per day**			
≤6	72	24	0.4
≥7	98	21	
**Level of education**			
Nil	37	6	0.07
Primary school	25	10	
Junior secondary school	33	7	
Senior secondary school	69	10	
Tertiary	6	12	
**Monthly salary in Naira**			
30,000–50,000 (USD ≤ 100)	124	10	0.043
60,000–100,000 (USD ≤ 200)	38	20	
>100,000 (USD ≥ 200)	8	15	
**Nature of work**			
Convertor	9	18	
Wood loader	27	0	
Sawmill supervisor	2	5	
Administrator	8	11	
Jackman	26	4	
Machine operator	44	3	0.05
Saw technician	13	8	
Sawyer	20	0	
Sawdust packer	21	0	
**Work experience in years**			
1−10	120	7	0.04
11−20	45	13	
21−30	5	15	
**Awareness of ocular hazards**			
Yes	131	38	0.03
No	39	7	
**Use of ocular safety devices**			
Yes	9	29	
No	161	16	0.02
**Type of ocular safety device**			
Goggles	5	15	0.7
Sunglasses	7	11	

## Data Availability

The datasets used and/or analyzed during the current study are available from the corresponding author on reasonable request.
